# Association of total, plant, and animal protein intake with hypertension among type 2 diabetic patients in Azar cohort population: A cross-sectional study

**DOI:** 10.34172/hpp.025.43378

**Published:** 2025-05-06

**Authors:** Sevil Kiani, Sina Naghshi, Elnaz Faramarzi, Maryam Saghafi-Asl

**Affiliations:** ^1^Student Research Committee, Tabriz University of Medical Sciences, Tabriz, Iran; ^2^Liver and Gastrointestinal Diseases Research Center, Tabriz University of Medical Sciences, Tabriz, Iran; ^3^Liver and Gastrointestinal Diseases Research Center, Department of Clinical Nutrition, Faculty of Nutrition and Food Sciences, Tabriz University of Medical Sciences, Tabriz, Iran

**Keywords:** Hypertension, Dietary proteins, Diabetes mellitus, Type 2, Cross-sectional studies

## Abstract

**Background:**

The existing research on the relationship between dietary protein intake and hypertension has mainly centered on the general population, with limited information available for adults with type 2 diabetes (T2DM). Therefore, this study was conducted to explore the association of total, plant, and animal protein intake with hypertension in adults with T2DM.

**Methods:**

In this cross-sectional study, 1947 individuals with T2DM from Azar cohort study were included. Dietary data were collected through a validated semi-quantitative food frequency questionnaire (FFQ). Hypertension was defined as blood pressure≥140/90 mm Hg, a self-reported diagnosis of hypertension confirmed by medical records, or the use of anti-hypertensive medications.

**Results:**

The mean age of the participants was 54.90 (SD: 8.25) years, with a majority (61%) being female. Initially, a significant positive relationship was observed between total protein intake and the odds of hypertension in the crude model (odds ratio [OR]: 1.38, 95% confidence interval [CI]: 1.04-1.83, *P*-trend: 0.055). However, after considering potential confounding factors, this association became non-significant (OR: 1.48, 95% CI: 0.94-2.33, *P*-trend: 0.224). Plant and animal protein intake did not show a significant association with hypertension, neither in the crude model nor in the fully-adjusted model. However, when participants were stratified by gender, a significant association was observed between total protein intake and hypertension only in men (OR: 2.52, 95% CI: 1.13-5.62, *P*-trend: 0.055).

**Conclusion:**

We found no evidence of an association between protein intake and hypertension among individuals with T2DM. However, in stratified analysis, there was a significant positive association between total protein intake and hypertension only in men. Future research should investigate potential, particularly sex-specific, mechanisms that may link dietary protein intake to hypertension in diverse populations.

## Introduction

 Hypertension is a prevalent public health concern worldwide, particularly among those with type 2 diabetes (T2DM). It is associated with an increased risk of cardiovascular disease and mortality.^1^ Among modifiable risk factors, diet plays an important role in the prevention and treatment of hypertension.^2^ The optimal composition of macronutrients to lower blood pressure remains a challenging question for researchers. This is particularly relevant for protein intake. In addition, protein subtypes, including plant and animal protein, may differentially affect the risk of hypertension.^3,4^ Findings from short-term randomized controlled trials (RCTs) suggest that substitution of protein for carbohydrates may lead to weight loss and lower blood pressure.^5,6^ However, extrapolation of such evidence to the whole or diseased populations needs to be done cautiously because RCTs have small sample sizes, substantial dropouts, short duration of intervention, and relatively large doses of protein intake.^7^ Observational studies are, therefore, of great importance in investigating association between protein intake and hypertension.

 There is a lack of evidence on the association between protein intake and hypertension among T2DM patients, and studies in the general population have provided conflicting findings.^8-10^ For instance, a Dutch cohort study of 3588 adults found no significant association between total, animal, and plant protein intake and hypertension risk.^8^ A cross-sectional study reported that increased animal protein intake in women was associated with reduced odds of hypertension, while no significant association was found for total and plant protein intake.^9^ Additionally, a longitudinal study documented an inverse relationship between animal protein intake and hypertension risk in both men and women, while plant protein intake was positively associated with hypertension risk among women.^10^ A study by Alonso et al^11^ indicated that a higher dietary intake of plant protein was significantly associated with a lower risk of hypertension, while no significant association was found for total and animal protein intake. These inconsistencies could be due to the variable amounts and types of protein intake in different populations. For example, in high-income countries, protein intake is high, especially from animal sources, while in low-income populations like Middle-Eastern countries, protein intake is limited and most of it comes from plant sources.^12,13^ Therefore, the findings of most available studies may not be applicable to low-income countries. The presence of unique characteristics in dietary intakes of Middle-Eastern countries warrants further studies, especially in diseased populations and can provide additional information for the relationship between protein intake and hypertension.^14^

 We hypothesized that the protein intake is associated with the prevalence of hypertension. Therefore, we designed a cross-sectional research using data from Azar cohort study to investigate the association of total, plant, and animal protein intake with hypertension in T2DM patients.

## Materials and Methods

###  Study design and participants

 Azar cohort study is an ongoing population-based cohort study conducted as part of the Prospective Epidemiological Research Studies in Iran (Persian cohort).^15-17^ This study began in October 2014 with the primary aim of investigating the key risk factors linked to non-communicable diseases. A comprehensive description of the study design has been previously documented,^15-17^ but in summary, around 15 000 adults between the ages of 35 and 70 years, who resided in Shabestar region for at least nine months, were recruited. For the current study, we used cross-sectional data from 2102 patients with T2DM who were identified based on a fasting blood sugar (FBS) concentration exceeding 126 mg/dl or self-reported previous diagnosis.^18,19^ We excluded pregnant women (n = 8) and those with a history of renal failure (n = 30), infectious diseases (n = 6), and type 1 diabetes mellitus (T1DM) (n = 11). T1DM was defined as insulin dependency with the onset before the age of 30.^20^ We further excluded those with missing data (n = 10) and those with daily energy intake below 800 kcal or above 4200 kcal^21^ (n = 90). Finally, a sample size of 1947 patients with T2DM remained for analysis. The study protocol was approved by the Ethics Committee of Tabriz University of Medical Sciences, Tabriz, Iran (IR.TBZMED.REC.1402.298). Written informed consent was obtained from all participants.

###  Assessment of dietary intake

 Dietary intakes were evaluated using a 130-item semi-quantitative food frequency questionnaire (FFQ), developed and validated specifically for Iranian adults.^22^ Trained interviewers administered the questionnaire and asked participants about their frequency of intake and portion size of each food item using images of a food portion or a predefined standard portion. After converting the food items into grams per day, the USDA Food Composition Tables (USDA-FCT) were used to obtain the average daily energy and nutrient intakes.^23^ Standard, non-branded foods from the USDA-FCT were selected for energy estimation. They were determined to closely resemble Iranian food items in terms of ingredients and macronutrients by four nutritionists. In cases of Iranian native foods, which were not present in the USDA-FCT, the energy content was estimated using the weighted average of major ingredients for that specific food item. Furthermore, the local food items were equated to standard FFQ items based on their major ingredients. Protein intake was expressed as a percentage of total energy intake. The main contributors to animal protein included processed and unprocessed red meat, fish, poultry, eggs, and dairy. Major dietary sources of plant protein included bread, cereals, pasta, nuts, beans, and legumes.

###  Assessment of blood pressure 

 After the participants had rested for at least 5 minutes, according to the Persian cohort protocol, blood pressure was measured twice in a sitting position.^17^ The patients rested for 10 minutes between each measurement. The average of the two measurements on each arm was considered for determining blood pressure of each participant. Hypertension was defined as meeting any of the following criteria: Systolic blood pressure (SBP) ≥ 140 mm Hg or diastolic blood pressure (DBP) ≥ 90 mm Hg, self-reported diagnosis of hypertension by a physician (with medical documents as proof), or the use of anti-hypertensive medications.^24,25^

###  Assessment of other variables 

 Data on general characteristics and lifestyle behaviors including gender, age, education level, marital status, medical history (including cardiovascular diseases, cancer and hypertension), medication history (including the use of anti-hypertensive, lipid-lowering, and blood glucose-lowering agents or insulin), duration of diabetes, smoking habits, alcohol consumption, and levels of physical activity were derived from pre-tested questionnaires. The level of physical activity was measured in terms of Metabolic Equivalent of Task (MET-hour per day). Height and weight were measured with light clothes and without shoes with an accuracy of 0.5 cm and 0.1 kg, respectively. Body mass index (BMI) was calculated by dividing weight (kg) by the square of height (m^2^). A non-stretchable measuring tape was used to measure waist circumference in accordance with the guidelines set by the National Institutes of Health (NIH).^15^

###  Statistical analysis

 Participants were divided into quintiles based on the percentage of protein intake from total daily energy. We applied one-way analysis of variance (ANOVA) for continuous variables and the chi-square test for categorical variables to compare the characteristics of study participants across the protein intake quintiles. Multiple logistic regression analysis was used to evaluate the association between protein intake and hypertension. The fully-adjusted model was controlled for potential confounding factors such as age (continuous), gender (male/female), energy intake (continuous), physical activity (continuous), marital status (single/married), education level (illiterate/university graduated/non-university education), duration of diabetes (continuous), smoking (yes/no), alcohol use (yes/no), medication use (yes/no), carbohydrates, saturated, monounsaturated, and polyunsaturated fatty acids (all in percentage of total energy), and BMI (continuous). Because BMI may be on the causal pathway, its inclusion in the model might be an over-adjustment.^26^ Therefore, we presented Model 1 without BMI and then, additionally adjusted for BMI in Model 2. To avoid issues of multicollinearity (assessed by variance inflation factor) among nutrients in regression analyses, we refrained from making additional adjustments. For all analyses, the bottom quintile of protein intake was used as the reference category. Also, the same set of covariates was used to examine the linear association per 3-percent increment in protein intake and odds of hypertension. Sensitivity analyses were performed after excluding alcohol consumers, cardiovascular and cancer patients, those using lipid-lowering drugs, and insulin users. We also excluded newly-diagnosed diabetic patients and repeated the analysis. These patients were defined to have FBS values more than 126 mg/dl with no prior diagnosis of diabetes.^18^ Additionally, we performed stratified analyses to examine potential risk modifiers such as age, gender, BMI, and smoking habits. These analyses allowed us to assess the impact of these factors on the relationship between protein intake and hypertension. All data analysis was conducted using IBM SPSS Statistics software version 19.0 (IBM Corp., Armonk, NY, USA) with a significance level of *P* < 0.05.

## Results

###  General patient characteristics and dietary intakes

 Patients were 54.90 (SD: 8.25) years old on average, and 61% were female. The overall prevalence of hypertension was 48.3% (39.1% in men and 54.3% in women). Median (interquartile range) intake of total, animal, and plant protein in the present study was 12.93% (11.96-14.02), 4.53% (3.58-5.76), and 8.23% (7.45-9.02) of total energy, respectively. Table 1 displays the general characteristics of the study participants based on the quintile of total, animal, and plant protein intake. Participants in the highest quintile of total protein intake were more likely to be male, university-educated, current smokers, and alcohol drinkers compared to those in the lowest quintile. In terms of plant protein, individuals in the top quintile had more physical activity and were more likely to be male than those in the lower quintile. Conversely, participants in the highest quintile of animal protein intake were less likely to be physically active and more likely to have a university education, drink alcohol, and take lipid-lowering medications than those in the lowest quintile. The dietary intakes of the participants across quintiles of total, plant, and animal protein intake can be found in Table 2. Participants with higher total protein intake tended to consume less carbohydrate, fat, and fruit but more cholesterol, vegetables, legumes, dairy, grains, meat, and micronutrients compared to those with lower intake. In terms of plant protein, those with higher intakes consumed less fat, but more carbohydrates and micronutrients. Higher intake of animal protein was associated with lower intake of carbohydrates and grains and higher intake of fat, cholesterol, micronutrients, meat, and dairy compared to lower intake. Participants in the highest quintile of plant protein consumed more legumes, vegetables, grains, and fiber, but less meat and dairy compared to those in the lowest quintile.

**Table 1:** General characteristics of the study participants across quintiles of total, animal, and plant protein intake

**Characteristics **	**Quintiles of total Protein**			**Quintiles of plant protein**			**Quintiles of animal protein**		
	**Quintile1** **N=391**	**Quintile3** **N=391 **	**Quintile5** **N=391 **	**Quintile1** **N=342 **	**Quintile3** **N=343 **	**Quintile5** **N=342 **	**Quintile1** **N=391**	**Quintile3** **N=392**	**Quintile5** **N=391**
Age (years)	54.4 (8.35)	55.4 (7.96)	54.8 (8.45)	55.7 (8.35)	54.9 (8.25)	54.5 (8.26)	54.4 (8.04)	54.9 (8.39)	54.9 (8.26)
Males (%)	113 (28.9)	165 (42.2)	177 (45.3)	123 (31.5)	154 (39.3)	172 (44.0)	133 (34.0)	165 (42.1)	153 (39.1)
Marital Status (married) (%)	348 (89.0)	348 (89.0)	359 (91.8)	346 (88.5)	351 (89.5)	353 (90.3)	341 (87.2)	357 (91.1)	346 (88.5)
Physical activity (MET/hour/day)	39.9 (6.55)	39.5 (7.00)	39.6 (7.19)	39.4 (6.60)	39.1 (6.27)	40.8 (8.58)	40.4 (7.35)	40.7 (8.28)	39.4 (6.65)
BMI (kg/m^2^)	30.9 (5.18)	30.4 (4.83)	30.0 (4.84)	30.3 (4.88)	30.4 (4.77)	30.4 (4.98)	30.9 (5.14)	30.2 (4.73)	30.1 (4.77)
Waist circumference (cm)	99.8 (10.5)	100 (10.1)	99.7 (10.9)	99.1 (10.6)	99.4 (10.3)	101 (10.7)	100 (10.5)	99.3 (10.0)	99.4 (11.0)
Smoking (smoker) (%)	43.0 (11.0)	41.0 (10.5)	52.0 (13.3)	49.0 (12.5)	54.0 (13.8)	46.0 (11.8)	40.0 (10.2)	47.0 (12.0)	45.0 (11.5)
Alcohol (alcoholic) (%)	4.00 (1.00)	4.00 (1.00)	8.00 (2.00)	7.00 (1.80)	9.00 (2.30)	3.00 (0.80)	4.00 (1.00)	3.00 (0.80)	10.0 (2.60)
Diabetes duration (year)	16.6 (21.0)	12.8 (17.5)	10.8 (14.8)	17.7 (21.8)	13.5 (18.2)	11.2 (15.6)	13.7 (18.6)	13.7 (18.9)	12.2 (16.7)
Education level (university graduated) (%)	16.0 (4.10)	24.0 (6.10)	28.0 (7.20)	18.0 (4.60)	20.0 (5.10)	16.0 (4.10)	13.0 (3.30)	21.0 (5.40)	28.0 (7.20)
CVD (yes) (%)	37.0 (9.50)	41.0 (10.5)	59.0 (15.1)	45.0 (11.5)	43.0 (11.0)	45.0 (11.5)	35.0 (9.00)	39.0 (9.90)	54.0 (13.8)
Lipid-lowering medication use (yes) (%)	80.0 (20.5)	112 (28.6)	123 (31.5)	98.0 (25.1)	98.0 (25.0)	101 (25.8)	89.0 (22.8)	90.0 (23.0)	130 (33.2)
Blood pressure-lowering medication use (yes) (%)	134 (34.3)	182 (46.5)	176 (45.0)	149 (38.1)	178 (45.4)	159 (40.7)	142 (36.3)	164 (41.8)	178 (45.5)
Glucose-lowering medication use (yes) (%)	238 (60.9)	296 (75.7)	318 (81.3)	250 (63.9)	295 (75.3)	318 (81.3)	272 (69.6)	283 (72.2)	308 (78.8)
Insulin use (yes) (%)	8.00 (2.00)	9.00 (2.30)	17.0 (4.30)	8.00 (2.00)	13.0 (3.30)	12.0 (3.10)	14.0 (3.60)	10.0 (2.60)	18.0 (4.60)
Newly-diagnosed diabetes (yes) (%)	97.0 (24.8)	56.0 (14.3)	39.0 (10.0)	100 (25.6)	66.0 (16.8)	45.0 (11.5)	71.0 (18.2)	70.0 (17.9)	50.0 (12.8)

Results obtained from One-way ANOVA (mean (SD)) or Chi-square (%), where appropriate. ANOVA, analysis of variance; SD, standard deviation; MET, metabolic equivalent of task; BMI, body mass index; CVD, cardiovascular disease.

**Table 2:** Dietary intakes of selected nutrients and food groups of the study participants across quintiles of total, animal, and plant protein intake

**Variables**	**Total protein**			**Plant protein**			**Animal protein**		
	**Quintile1** **N=391 **	**Quintile 3** **N=391**	**Quintile5** **N=391**	**Quintile1** **N=342**	**Quintile 3** **N=343**	**Quintile5** **N=342**	**Quintile1** **N=391**	**Quintile 3** **N=392**	**Quintile5** **N=391**
Nutrients									
Energy (kcal)	2437 (652)	2452 (695)	2414 (706)	2387 (666)	2464 (644)	2447 (704)	2458 (696)	2444 (651)	2379 (695)
Carbohydrate (% energy)	61.2 (6.21)	60.9 (4.94)	57.6 (4.96)	59.0 (6.29)	62.4 (4.63)	64.9 (4.16)	65.6 (5.25)	62.3 (4.60)	58.0 (4.85)
Fat (% energy)	28 (6.19)	26.2 (5.11)	27 (4.71)	31.9 (5.77)	27.2 (4.09)	23.5 (3.70)	25.4 (5.70)	27.5 (5.09)	29.6 (4.82)
Cholesterol (mg)	223 (91.6)	236 (95.9)	301 (126)	289 (118)	250 (96.4)	204 (84.5)	189 (86.5)	250 (88.0)	317 (126)
Dietary fiber (g)	31.4 (9.57)	31.5 (9.69)	31.8 (9.61)	30.0 (9.42)	32.3 (9.32)	32.5 (9.92)	31.8 (10.0)	31.8 (8.72)	30.6 (9.28)
Potassium (mg)	4027 (1146)	4023 (1186)	4335 (1244)	4206 (1200)	4186 (1156)	3927 (1199)	3861 (1204)	4092 (1060)	4339 (1259)
Sodium (mg)	3519 (1136)	3917 (1310)	3849 (1275)	3439 (1259)	3776 (1132)	4048 (1229)	3876 (1271)	3775 (1101)	3685 (1288)
Zinc (mg)	8.88 (2.41)	10.1 (2.85)	11.7 (3.36)	9.91 (3.09)	10.3 (2.82)	10.3 (3.01)	9.09 (2.68)	10.1 (2.72)	11.4 (3.42)
Phosphor (mg)	1123 (293)	1282 (348)	1438 (388)	1240 (361)	1299 (337)	1302 (373)	1151 (332)	1283 (326)	1409 (393)
Iron (mg)	15.6 (4.65)	18.1 (5.86)	18.7 (5.87)	14.3 (4.30)	17.6 (4.82)	20.8 (6.16)	18.4 (5.90)	17.6 (5.31)	16.7 (5.33)
Magnesium (mg)	358 (95.1)	377 (104)	401 (113)	360 (99.4)	383 (99.3)	391 (112)	366 (104)	377 (96.0)	386 (109)
Calcium (mg)	1033 (292)	1240 (375)	1294 (385)	1102 (353)	1211 (332)	1314 (396)	1142 (359)	1232 (354)	1236 (374)
Food groups (g/d)									
Grains	407 (163)	473 (200)	424 (187)	326 (141)	447 (151)	541 (197)	503 (191)	442 (173)	369 (167)
Fruits	705 (347)	572 (320)	526 (276)	681 (326)	621 (287)	463 (258)	597 (348)	599 (300)	566 (285)
Vegetables	536 (214)	568 (255)	621 (278)	549 (245)	592 (256)	584 (263)	535 (234)	567 (231)	609 (291)
Nuts	9.43 (9.11)	9.10 (9.73)	9.68 (9.13)	10.2 (9.30)	9.97 (9.33)	7.94 (8.77)	8.07 (9.02)	9.34 (9.89)	10.2 (8.64)
Legumes	24.1 (15.0)	29.2 (19.3)	39.8 (33.1)	22.3 (14.4)	30.4 (20.0)	41.2 (33.8)	28.5 (20.5)	32.5 (27.7)	31.8 (24.7)
Dairy	260 (143)	344 (185)	429 (231)	422 (233)	354 (183)	275 (158)	208 (112)	362 (156)	469 (239)
Meat	33.4 (22.6)	47.6 (26.5)	91.5 (50.0)	63.7 (45.8)	56.2 (34.9)	40.3 (27.1)	25.0 (15.8)	48.9 (23.0)	96.2 (48.7)

Results obtained from One-way ANOVA (mean (SD)). ANOVA, analysis of variance; SD, standard deviation.

###  Association between protein intake and hypertension

Table 3 presents the crude and multivariable-adjusted odds ratios (ORs) and 95% confidence intervals (CIs) for the association between total, plant, and animal protein intake and hypertension. A positive significant association was observed between total protein intake and the odds of hypertension in the crude model (OR: 1.38, 95% CI: 1.04-1.83, *P*-trend: 0.055). However, after adjusting for potential confounders, this positive association was no longer significant (OR: 1.48, 95% CI: 0.94-2.33, *P*-trend: 0.224). Neither the crude model (OR: 1.10, 95% CI: 0.83-1.46, *P*-trend: 0.305), nor the fully-adjusted model (OR: 1.26, 95% CI: 0.81-1.94, *P*-trend: 0.212) showed a significant association between plant protein intake and hypertension. Similarly, there was no significant association between higher intakes of animal protein and the likelihood of hypertension either before (OR: 1.23, 95% CI: 0.93-1.63, *P*-trend: 0.086) or after (OR: 1.11, 95% CI: 0.77-1.60, *P*-trend: 0.496) controlling for potential confounders. Additionally, when protein intakes were modeled continuously, each 3-percent increase in protein intake did not show a significant association with hypertension after adjusting for confounders.

**Table 3:** Multivariable-adjusted odds ratios for hypertension across quintiles of total, animal, and plant protein intake

	**Total population**						
	**Quintile1**	**Quintile 2**	**Quintile 3**	**Quintile 4**	**Quintile 5**	**P-trend**	**Per 3-percent increase in protein intake**
			**Total protein**				
Intake category (% energy)	< 11.7	11.7-12.5	12.5-13.3	13.3-14.2	> 14.2		
Participants/Cases	391/165	387/190	390/207	389/188	390/196		
Crude model	1	1.31 (0.99, 1.74)	1.55 (1.17, 2.06)	1.27 (0.96, 1.69)	1.38 (1.04, 1.83)	0.055	1.16 (0.99, 1.36)
Model I ^a^	1	1.27 (0.91, 1.75)	1.55 (1.09, 2.22)	1.28 (0.87, 1.88)	1.37 (0.87, 2.13)	0.296	1.10 (0.82, 1.48)
Model II ^b^	1	1.34 (0.96, 1.87)	1.63 (1.13, 2.34)	1.32 (0.89, 1.94)	1.48 (0.94, 2.33)	0.224	1.14 (0.85, 1.54)
			**Plant protein**				
Intake category (% energy)	< 7.2	7.2-7.9	7.9-8.5	8.5-9.2	> 9.2		
Participants/Cases	390/179	389/184	390/198	387/196	391/189		
Crude model	1	1.05 (0.79, 1.40)	1.21 (0.91, 1.61)	1.21 (0.91, 1.60)	1.10 (0.83, 1.46)	0.305	1.06 (0.84, 1.32)
Model I ^a^	1	1.18 (0.85, 1.62)	1.41 (1.00, 1.99)	1.41 (0.97, 2.04)	1.30 (0.85, 2.00)	0.155	1.11 (0.76, 1.61)
Model II ^b^	1	1.16 (0.84, 1.61)	1.39 (0.98, 1.97)	1.38 (0.95, 2.02)	1.26 (0.81, 1.94)	0.212	1.09 (0.75, 1.59)
			**Animal protein**				
Intake category (% energy)	< 3.3	3.3-4.1	4.1-5.0	5.0-6.1	> 6.1		
Participants/Cases	390/179	388/183	391/189	389/196	389/199		
Crude model	1	1.05 (0.79, 1.39)	1.10 (0.83, 1.46)	1.19 (0.90, 1.58)	1.23 (0.93, 1.63)	0.086	1.12 (0.96, 1.31)
Model I ^a^	1	1.01 (0.74, 1.38)	1.08 (0.79, 1.48)	1.09 (0.79, 1.50)	1.05 (0.73, 1.50)	0.660	1.01 (0.82, 1.25)
Model II ^b^	1	1.06 (0.77, 1.45)	1.12 (0.81, 1.54)	1.13 (0.81, 1.56)	1.11 (0.77, 1.60)	0.496	1.04 (0.83, 1.29)

^a^ Adjusted for age, energy intake, gender, education status, smoking status, alcohol use, marital status, physical activity, medication use, diabetes duration, carbohydrate intake (%), saturated fat (%), monounsaturated fat (%), and polyunsaturated fat (%). ^b^ Adjusted for age, energy intake, gender, education status, smoking status, alcohol use, marital status, physical activity, medication use, diabetes duration, carbohydrate intake (%), saturated fat (%), monounsaturated fat (%), polyunsaturated fat (%), and BMI.

###  Sensitivity and stratified analyses

 The associations between protein intake and hypertension remained robust in the fully-adjusted models across sensitivity and stratified analyses (Supplementary file 1, Table S1 and S2). However, upon stratification by gender in the fully-adjusted model, a significant positive association was observed between total protein intake and odds of hypertension in men (OR: 2.52, 95% CI: 1.13-5.62, *P*-trend: 0.055), while no significant association was found in women. Furthermore, associations of plant and animal protein intake with the likelihood of hypertension remained non-significant in fully-adjusted models for both men and women (Table S2).

## Discussion

 In the present large cross-sectional study of T2DM patients, we found no significant associations between higher intake of total, plant, and animal proteins and the odds of hypertension. A similar pattern of associations was observed across sensitivity and stratified analyses except for total protein intake and hypertension in men, where higher total protein intake was associated with greater odds of hypertension. To the best of our knowledge, this study appears to be the first to examine the association of protein intake with hypertension among a large sample of T2DM patients in the Middle East region.

 Hypertension poses a significant global health concern, particularly among individuals with T2DM. The simultaneity of hypertension and T2DM is associated with an increased risk of cardiovascular diseases.^1^ Therefore, the prevention and management of hypertension in diabetic patients is important. While the available evidence suggests that diet can affect blood pressure, there is still uncertainty about the relevance of total, plant, and animal protein intake.^2^ Unlike Western countries, plant-based foods are the major source of protein intake in Asian countries, especially Iran. Therefore, the patterns of associations between total, animal, and plant protein intake and hypertension in such populations are probably different from those in Western countries. Furthermore, it is well known that the metabolism of carbohydrates and other macronutrients is different in diabetic patients compared to the general population.^27^ Insulin resistance is a key feature of patients with T2DM, leading to elevated blood glucose levels by disrupting carbohydrate metabolism and impairing the body’s ability to effectively use glucose. This condition forces the body to rely more on fat for energy, which can result in dyslipidemia. Additionally, insulin resistance impacts protein metabolism by hindering the cellular uptake of amino acids and protein synthesis.^28,29^ Therefore, diet-disease associations in people with diabetes may differ from that in other people.

 In the current study, total, animal, and plant proteins were not associated with the odds of hypertension. Findings from the Rotterdam study also documented no significant relationship between total, animal, and plant proteins and the risk of hypertension over 6 years.^30^ Similarly, a prospective cohort study involving European individuals with T1DM reported no significant associations between total, animal, or plant protein intake and the incidence of hypertension during a 7-year follow-up period.^31^ Another study with a cross-sectional design involving 518 participants also reported a non-significant association between protein intake and hypertension.^32^ A cohort study in Japan also concluded that total protein intake was not associated with hypertension risk.^33^ Furthermore, a study conducted in the general Dutch population found no significant links between total, plant, or animal protein intake and the risk of hypertension ^8^. In terms of RCTs, a meta-analysis in which study durations ranged from 4 to 24 weeks reported significant favorable effects of high-protein diets compared with control diets on blood pressure in T2DM patients.^34^ However, the findings of another meta-analysis that included only studies with an intervention duration of 12 weeks or more showed that a high-protein diet had no significant effect on blood pressure levels among T2DM patients.^35^ Overall, the findings from some RCTs should be interpreted with caution because they cannot reliably assess realistic and long-term effects of high-protein diets on blood pressure levels.^7^

 The study of Matos et al^36^ provided different findings and showed that increased consumption of total protein and meat was positively associated with uncontrolled daytime blood pressure in patients with T2DM. Discrepancies could arise from differences in dietary and blood pressure assessment methods and criteria for defining uncontrolled blood pressure. In their study, the usual diet was assessed using 3-day weighed diet records, blood pressure was assessed using office measurements and 24-hour ambulatory blood pressure monitoring, and hypertension was defined as blood pressure ≥ 135/85 mm Hg.

 An analysis of the Framingham Heart Study Offspring cohort revealed that higher total and animal protein intake, but not plant protein intake, was beneficially associated with annualized changes in SBP.^37^ However, compared to ours, mixed models were used for data analysis in their study and the relationship between protein consumption and SBP and DBP was separately examined.

 The study by Jamshidi et al^38^ demonstrated that increased intake of total and plant protein was inversely associated with the odds of hypertension, while no significant association was found for animal protein intake. The investigators in their study used quintile of protein intake based on the amount of grams per kilogram of body weight. Moreover, the sample size was larger compared to ours, which can increase the power of their study in finding significant associations.

 A longitudinal study found that animal protein intake was inversely associated with hypertension risk, total protein intake had a non-significant association, and plant protein intake was positively associated with hypertension among women.^10^ Another study suggested that higher intakes of animal protein in women, but not in men, were related to lower odds of hypertension, while no significant association was found for total and plant protein intakes.^9^

 Several differences between our study and the previous ones could explain the discrepancies between the results. First, we investigated diabetic patients while previous studies were conducted on general population. Second, there were differences in the amount and type of protein intake among studies. Median (interquartile range) intake of total, animal, and plant protein in the present study was 12.93% (11.96-14.02), 4.53% (3.58-5.76), and 8.23% (7.45-9.02) of total energy, respectively. This indicates that plant proteins constitute the majority of protein intake in our study, contrasting with findings from other studies where individuals typically consume more animal proteins than plant proteins. For example, in the study conducted by Altorf-van der Kuil et al,^31^ the mean protein intake was reported as 17.6% for total protein, with 12.3% from animal sources and 5.2% from plant sources. In the study conducted by Mattos et al,^36^ individuals with uncontrolled blood pressure had an average protein intake of 20% of their total energy, while those with controlled blood pressure had a slightly lower average of 18.2%. In another investigation, 60.6% of total protein was derived from animal sources.^30^ Third, in the studies of Liu et al^9^ and He et al,^10^ protein intake was energy-adjusted using the residual method and expressed as grams per day, while in our study, the nutrient density method was used and protein was expressed as a percentage of total energy intake. Fourth, different methods used in the processing and cooking of protein-rich foods and their biologically active components were not considered in statistical analyses which can provide further explanation for this discrepancy.

 In a further analysis of men and women separately, a positive association between total protein intake and hypertension was evident in men, but not in women. To justify this finding, it can be pointed out that the range of protein intake was narrower in women compared to men. Thus, the small variations in total protein intake among women in our study may have led to such non-significant finding. A comparison of the highest and lowest intakes of meat in total protein categories in men and women revealed significantly higher and wider quantities consumed by men compared to women. Moreover, sex hormones may play a specific role in the development and progression of diseases; however, the exact molecular mechanisms involved in these physiological processes are not yet fully understood.^39^

 In terms of individual food sources of protein, some reports have documented that red and processed meat^40^ and refined grains^41^ increase the risk of hypertension, while legumes,^42,43^ nuts,^40^ and dairy products^40^ are associated with a decrease in the incidence of hypertension. It should be kept in mind that in such reports, the exposure variable was a food group, while in our study, the exposure variable was protein as a nutrient. In addition to protein, food groups contain other compounds such as vitamins, minerals, fats, carbohydrates, and additives, which in turn can affect the risk of hypertension.. Therefore, it is reasonable that the findings obtained for food groups and protein are different.

 The precise mechanisms by which dietary protein affects blood pressure remain largely unknown. The amino acid composition of proteins may partially have contributed to their effect on blood pressure. For example, arginine, which acts through nitric oxide, has been shown to lower blood pressure and enhance endothelial function.^44^ Long-term supplementation with taurine has also shown anti-hypertensive effects.^45^ Moreover, aromatic amino acids serve as precursors for serotonin (from tryptophan) and catecholamines such as dopamine, norepinephrine, and epinephrine (from phenylalanine and tyrosine) play crucial roles in the sympathetic nervous system.^46^ Activation of the sympathetic system can raise blood pressure by increasing heart rate and constricting blood vessels through catecholamines. Elevated sympathetic nerve activity, along with higher levels of norepinephrine and epinephrine, have been linked to hypertension.^47^ Moreover, certain byproducts resulting from bacterial breakdown of proteins and amino acids in the intestinal tract may be associated with hypertension.^48^ It should also be noted that previous studies have shown inconsistent associations between individual amino acids and hypertension.^49^ Therefore, the overall relationship between protein intake and high blood pressure seems to be a result of the interaction of amino acids with each other and with the gut microbiota.

 The present study has several strengths. It is the first study to investigate the link between protein intake and hypertension among a large sample of T2DM patients. Controlling for major risk factors of hypertension, performing several subgroup and sensitivity analyses to examine the robustness of the findings, and investigating the relationships based on the protein subtypes are further strengths of this study. However, it is essential to acknowledge the limitations of our study. Firstly, unmeasured confounding variables may have influenced the strength of the relationship between protein intake and hypertension. Secondly, diets rich in plant and animal proteins might be associated with healthier and less healthy lifestyles, respectively, which may not have been accurately controlled in our analysis. Thirdly, the cross-sectional nature of the study prevents establishing causal or temporal relationships between the variables studied. Additional longitudinal studies are needed to clarify the exact association between dietary protein intake and blood pressure. Fourthly, measurement errors in dietary assessments could have led to underestimations of the investigated associations. Lastly, our study was conducted among adults with T2DM, and therefore, our findings are less generalizable to healthy populations and patients with other metabolic diseases.

## Conclusion

 In conclusion, our study did not identify any significant associations between the intake of total, plant, and animal proteins and odds of hypertension in T2DM patients. These findings have potentially public health implications, as they do not support a potential benefit from the inclusion of protein in T2DM patients. As a take-home message, higher protein intake in diabetic patients may not lower blood pressure. Considering the cross-sectional nature of the study, the findings should be interpreted with caution and larger-scale prospective studies are needed to confirm our findings.

## Competing Interests

 The authors declare no conflict of interest.

## Ethical Approval

 The research followed the guidelines outlined in the Declaration of Helsinki, and all protocols involving human participants were approved by the Bioethics Committee of Tabriz University of Medical Sciences, Tabriz, Iran (Ethics Number: IR.TBZMED.REC.1402.298).

## Supplementary Files

Supplementary file 1 contains Table S1 and S2.
