# Supplementary material for: Association of total, plant, and animal protein intake with hypertension among type 2 diabetic patients in Azar cohort population: A cross-sectional study
Source: Health Promot Perspect. 2025 May 6;15(1):54–62. doi: 10.34172/hpp.025.43378 (PMC12125505; doi:10.34172/hpp.025.43378)
Supplement: Supplementary file 1 — contains Table S1 and S2. [file hpp-15-54-s001.pdf]

Kiani et al, Health Promotion Perspectives. 2025;15(1):54-S1
doi: 10.34172/hpp.025.43378
https://hpp.tbzmed.ac.ir

Supplementary file 1

Table s1. Sensitivity analyses for the association between total, animal, and plant protein intake and odds of hypertension.

**Excluding CVD and cancer patients**							
	**Quintile1**	**Quintile 2**	**Quintile 3**	**Quintile 4**	**Quintile 5**	**P-trend**	**Per 3-percent increase in protein intake**
**Total protein**							
Participants	342	340	342	341	342		
Fully-adjusted Model [1]	1	1.40 (0.98, 2.00)	1.62 (1.10, 2.39)	1.31 (0.86, 2.00)	1.54 (0.94, 2.51)	0.239	1.11 (0.81, 1.54)
**Plant protein**							
Participants	342	341	341	341	342		
Fully-adjusted Model [1]	1	1.16 (0.82, 1.65)	1.42 (0.98, 2.06)	1.39 (0.93, 2.08)	1.24 (0.78, 1.97)	0.266	1.08 (0.72, 1.61)
**Animal protein**							
Participants	342	341	341	341	342		
Fully-adjusted Model [1]	1	1.00 (0.71, 1.40)	1.19 (0.85, 1.68)	1.28 (0.90, 1.82)	1.14 (0.77, 1.69)	0.212	1.03 (0.81, 1.29)
**Excluding consumers of lipid-lowering agents and insulin**							
	**Quintile1**	**Quintile 2**	**Quintile 3**	**Quintile 4**	**Quintile 5**	**P-trend**	**Per 3-percent increase in protein intake**
**Total protein**							
Participants	280	278	277	279	278		
Fully-adjusted Model [2]	1	1.50 (1.01, 2.22)	1.80 (1.17, 2.78)	1.23 (0.77, 1.98)	1.61 (0.93, 2.78)	0.364	1.23 (0.86, 1.76)
**Plant protein**							
Participants	279	278	279	276	280		
Fully-adjusted Model [2]	1	1.27 (0.86, 1.87)	1.45 (0.95, 2.19)	1.36 (0.86, 2.15)	1.53 (0.91, 2.60)	0.146	1.24 (0.79, 1.96)
**Animal protein**							
Participants	279	278	279	278	278		
Fully-adjusted Model [2]	1	1.16 (0.80, 1.70)	1.17 (0.80, 1.72)	1.11 (0.75, 1.64)	1.25 (0.81, 1.92)	0.430	1.03 (0.80, 1.34)
**Excluding newly-diagnosed diabetic patients**							

	Quintile1	Quintile 2	Quintile 3	Quintile 4	Quintile 5	P-trend	Per 3-percent increase in protein intake
**Total protein**							
Participants	327	324	327	326	326		
Fully-adjusted Model [1]	1	1.18 (0.83, 1.69)	1.16 (0.79, 1.71)	1.27 (0.84, 1.93)	1.18 (0.72, 1.92)	0.477	1.10 (0.80, 1.52)
**Plant protein**							
Participants	326	326	327	324	327		
Fully-adjusted Model [1]	1	1.00 (0.70, 1.42)	1.33 (0.91, 1.93)	1.14 (0.76, 1.71)	0.91 (0.57, 1.45)	0.981	0.92 (0.61, 1.39)
**Animal protein**							
Participants	326	326	327	325	326		
Fully-adjusted Model [1]	1	1.02 (0.73, 1.43)	1.10 (0.78, 1.56)	1.14 (0.80, 1.63)	1.10 (0.74, 1.65)	0.460	1.08 (0.85, 1.36)

Excluding current and former alcohol drinkers

	Quintile1	Quintile 2	Quintile 3	Quintile 4	Quintile 5	P-trend	Per 3-percent increase in protein intake
**Total protein**							
Participants	358	356	357	358	358		
Fully-adjusted Model [3]	1	1.37 (0.97, 1.93)	1.51 (1.04, 2.19)	1.19 (0.80, 1.78)	1.42 (0.88, 2.28)	0.421	1.12 (0.82, 1.53)
**Plant protein**							
Participants	358	357	358	355	359		
Fully-adjusted Model [3]	1	1.19 (0.85, 1.67)	1.36 (0.95, 1.95)	1.33 (0.90, 1.96)	1.19 (0.76, 1.86)	0.386	1.08 (0.73, 1.60)
**Animal protein**							
Participants	358	357	358	357	357		
Fully-adjusted Model [3]	1	0.98 (0.71, 1.36)	1.00 (0.71, 1.39)	1.05 (0.75, 1.47)	1.11 (0.76, 1.63)	0.526	1.03 (0.82, 1.29)

[1] Adjusted for age, energy intake, gender, education status, smoking status, alcohol use, marital status, physical activity, medication use, diabetes duration, carbohydrate intake (%), saturated fat (%), monounsaturated fat (%), polyunsaturated fat (%), and BMI.

[2] Adjusted for age, energy intake, gender, education status, smoking status, alcohol use, marital status, physical activity, diabetes duration, carbohydrate intake (%), saturated fat (%), monounsaturated fat (%), polyunsaturated fat (%), and BMI.

[3] Adjusted for age, energy intake, gender, education status, smoking status, marital status, physical activity, medication use, diabetes duration, carbohydrate intake (%), saturated fat (%), monounsaturated fat (%), polyunsaturated fat (%), and BMI.

CVD, cardiovascular disease.

Table S2. Subgroup analyses for the association between total, animal, and plant protein intake and odds of hypertension.

	Quintile1	Quintile 2	Quintile 3	Quintile 4	Quintile 5	P-trend	Per 3-percent increase in protein intake
**Age≥60 years**							
**Total protein**							
Participants	120	123	130	117	123		
Fully-adjusted Model [1]	1	2.07 (1.14, 3.73)	2.14 (1.12, 4.09)	2.04 (1.01, 4.13)	2.19 (0.98, 4.91)	0.144	1.43 (0.85, 2.40)
**Plant protein**							
Participants	145	114	118	120	115		
Fully-adjusted Model [1]	1	1.71 (0.96, 3.03)	1.96 (1.05, 3.63)	2.30 (1.17, 4.51)	1.95 (0.89, 4.23)	0.069	1.68 (0.83, 3.38)
**Animal protein**							
Participants	111	112	122	140	127		
Fully-adjusted Model [1]	1	1.09 (0.60, 1.95)	1.25 (0.69, 2.26)	1.04 (0.57, 1.87)	1.29 (0.67, 2.48)	0.573	1.03 (0.70, 1.50)
**Age<60 years**							
	**Quintile1**	**Quintile 2**	**Quintile 3**	**Quintile 4**	**Quintile 5**	**P-trend**	**Per 3-percent increase in protein intake**
**Total protein**							
Participants	271	265	259	273	267		
Fully-adjusted Model [1]	1	1.21 (0.81, 1.79)	1.60 (1.04, 2.46)	1.23 (0.78, 1.95)	1.49 (0.87, 2.58)	0.252	1.14 (0.79, 1.64)
**Plant protein**							
Participants	245	275	272	267	276		
Fully-adjusted Model [1]	1	0.92 (0.62, 1.37)	1.15 (0.76, 1.74)	1.02 (0.65, 1.60)	0.96 (0.57, 1.60)	0.948	0.86 (0.55, 1.33)
**Animal protein**							
Participants	279	276	269	249	262		
Fully-adjusted Model [1]	1	1.05 (0.73, 1.51)	1.10 (0.76, 1.60)	1.29 (0.87, 1.90)	1.13 (0.73, 1.75)	0.313	1.12 (0.87, 1.46)
**BMI≥25 Kg/m2**							
	**Quintile1**	**Quintile 2**	**Quintile 3**	**Quintile 4**	**Quintile 5**	**P-trend**	**Per 3-percent increase in protein intake**
**Total protein**							
Participants	351	343	345	344	338		

Fully-adjusted Model [2]	1	1.25 (0.89, 1.77)	1.54 (1.05, 2.24)	1.28 (0.86, 1.92)	1.31 (0.81, 2.10)	0.378	1.08 (0.79, 1.48)
**Plant protein**							
Participants	341	347	347	338	348		
Fully-adjusted Model [2]	1	1.16 (0.82, 1.63)	1.47 (1.02, 2.12)	1.40 (0.94, 2.08)	1.33 (0.84, 2.09)	0.154	1.15 (0.78, 1.72)
**Animal protein**							
Participants	355	338	344	347	337		
Fully-adjusted Model [2]	1	1.06 (0.77, 1.47)	1.11 (0.80, 1.55)	1.07 (0.76, 1.50)	0.96 (0.66, 1.42)	0.976	0.99 (0.79, 1.24)
**BMI<25 kg/m2**							
	**Quintile1**	**Quintile 2**	**Quintile 3**	**Quintile 4**	**Quintile 5**	**P-trend**	**Per 3-percent increase in protein intake**
**Total protein**							
Participants	40	45	44	46	52		
Fully-adjusted Model [2]	1	1.35 (0.42, 4.28)	1.85 (0.53, 6.39)	1.45 (0.37, 5.58)	2.40 (0.51, 11.0)	0.333	1.53 (0.58, 4.03)
**Plant protein**							
Participants	49	42	43	49	43		
Fully-adjusted Model [2]	1	1.03 (0.35, 3.05)	0.77 (0.24, 2.44)	1.14 (0.34, 3.84)	0.72 (0.16, 3.21)	0.807	0.61 (0.17, 2.21)
**Animal protein**							
Participants	35	50	47	42	52		
Fully-adjusted Model [2]	1	1.21 (0.39, 3.71)	1.34 (0.41, 4.33)	1.80 (0.51, 6.32)	3.61 (1.00, 13.0)	0.033	1.47 (0.71, 3.01)
**Smokers**							
	**Quintile1**	**Quintile 2**	**Quintile 3**	**Quintile 4**	**Quintile 5**	**P-trend**	**Per 3-percent increase in protein intake**
**Total protein**							
Participants	43	44	41	53	52		
Fully-adjusted Model [3]	1	0.49 (0.14, 1.77)	0.12 (0.22, 0.66)	0.32 (0.06, 1.60)	0.42 (0.06, 2.68)	0.824	0.79 (0.23, 2.73)
**Plant protein**							
Participants	49	38	54	45	46		
Fully-adjusted Model [3]	1	0.61 (0.17, 2.13)	0.81 (0.25, 2.61)	0.91 (0.24, 3.34)	0.86 (0.19, 3.81)	0.933	0.89 (0.25, 3.16)
**Animal protein**							
Participants	40	48	47	52	45		
Fully-adjusted Model [3]	1	0.62 (0.18, 2.10)	0.58 (0.17, 1.99)	0.91 (0.26, 3.10)	0.46 (0.11, 1.86)	0.553	0.93 (0.40, 2.16)

	Quintile1	Quintile 2	Quintile 3	Quintile 4	Quintile 5	P-trend	Per 3-percent increase in protein intake
**Non-smokers**							
**Total protein**							
Participants	348	344	348	337	338		
Fully-adjusted Model [3]	1	1.39 (0.98, 1.97)	1.91 (1.31, 2.79)	1.40 (0.93, 2.10)	1.47 (0.91, 2.37)	0.219	1.14 (0.83, 1.56)
**Plant protein**							
Participants	341	351	336	342	345		
Fully-adjusted Model [3]	1	1.27 (0.90, 1.78)	1.48 (1.02, 2.14)	1.53 (1.03, 2.28)	1.40 (0.89, 2.22)	0.107	1.19 (0.80, 1.78)
**Animal protein**							
Participants	350	340	344	337	344		
Fully-adjusted Model [3]	1	1.06 (0.77, 1.48)	1.17 (0.83, 1.63)	1.09 (0.77, 1.54)	1.15 (0.78, 1.69)	0.485	1.01 (0.80, 1.27)
**Men**							
	**Quintile1**	**Quintile 2**	**Quintile 3**	**Quintile 4**	**Quintile 5**	**P-trend**	**Per 3-percent increase in protein intake**
**Total protein**							
Participants	113	126	165	180	177		
Fully-adjusted Model [4]	1	1.68 (0.90, 3.13)	1.58 (0.82, 3.04)	1.77 (0.89, 3.52)	2.52 (1.13, 5.62)	0.055	1.59 (0.94, 2.67)
**Plant protein**							
Participants	123	141	154	170	172		
Fully-adjusted Model [4]	1	1.38 (0.77, 2.49)	1.61 (0.87, 2.96)	1.62 (0.84, 3.11)	1.95 (0.91, 4.19)	0.113	1.44 (0.74, 2.81)
**Animal protein**							
Participants	132	146	165	164	153		
Fully-adjusted Model [4]	1	1.01 (0.58, 1.76)	1.27 (0.74, 2.18)	1.46 (0.84, 2.52)	1.11 (0.58, 2.09)	0.335	1.12 (0.77, 1.63)
**Women**							
	**Quintile1**	**Quintile 2**	**Quintile 3**	**Quintile 4**	**Quintile 5**	**P-trend**	**Per 3-percent increase in protein intake**
**Total protein**							
Participants	278	262	224	210	213		

Fully-adjusted Model [4]	1	1.17 (0.78, 1.73)	1.81 (1.15, 2.84)	1.12 (0.69, 1.81)	1.06 (0.60, 1.88)	0.860	0.97 (0.67, 1.41)
**Plant protein**							
Participants	267	248	236	217	219		
Fully-adjusted Model [4]	1	1.11 (0.74, 1.65)	1.28 (0.83, 1.97)	1.35 (0.84, 2.17)	1.03 (0.60, 1.77)	0.631	1.00 (0.63, 1.58)
**Animal protein**							
Participants	258	242	226	225	236		
Fully-adjusted Model [4]	1	1.10 (0.75, 1.63)	1.02 (0.68, 1.53)	0.91 (0.60, 1.37)	1.09 (0.69, 1.73)	0.944	0.98 (0.75, 1.29)

[1] Adjusted for energy intake, gender, education status, smoking status, alcohol use, marital status, physical activity, medication use, diabetes duration, carbohydrate intake (%), saturated fat (%), monounsaturated fat (%), polyunsaturated fat (%), and BMI.

[2] Adjusted for age, energy intake, gender, education status, smoking status, alcohol use, marital status, physical activity, diabetes duration, carbohydrate intake (%), saturated fat (%), monounsaturated fat (%), polyunsaturated fat (%), and medication use.

[3] Adjusted for age, energy intake, gender, education status, alcohol use, marital status, physical activity, medication use, diabetes duration, carbohydrate intake (%), saturated fat (%), monounsaturated fat (%), polyunsaturated fat (%), and BMI.

[4] Adjusted for age, energy intake, education status, smoking status, alcohol use, marital status, physical activity, medication use, diabetes duration, carbohydrate intake (%), saturated fat (%), monounsaturated fat (%), polyunsaturated fat (%), and BMI.

BMI, body mass index.
